# Improvements in Clinical Cancer Care Associated with Integration of Personalized Medicine

**DOI:** 10.3390/jpm14090997

**Published:** 2024-09-20

**Authors:** Arushi Agarwal, Daryl Pritchard, Alissa Winzeler, Hina Mohammed, Thomas D. Brown, Gary G. Gustavsen

**Affiliations:** 1 Health Advances LLC, San Francisco, CA 94105, USA; 2 Personalized Medicine Coalition, Washington, DC 20036, USA; dpritchard@personalizedmedicinecoalition.org; 3 Syapse, West Chester, PA 19380, USA; awinzeler@privatehealth.com (A.W.); hina.mohammed@syapse.com (H.M.); tom.brown@syapse.com (T.D.B.); 4 Private Health Management, Los Angeles, CA 90024, USA; 5 Health Advances LLC, Newton, MA 04266, USA; ggustavsen@healthadvances.com

**Keywords:** precision medicine, personalized medicine, maturity model, targeted therapies, clinical trials

## Abstract

Background: While adoption of personalized medicine (PM) continues to increase in clinical oncology, there is limited data connecting the level of PM adoption at a given institution to improved clinical outcomes for patients. The purpose of this study was to analyze the correlation between health care providers’ scores on a previously described PM integration framework and two outcome measures: the use of targeted therapy and clinical trial enrollment. Methods: This study was conducted using real-world data (RWD) from the Syapse^®^ Learning Health Network (LHN). The PM integration score for six community hospital systems in the LHN was calculated and subsequently correlated with the two outcome measures. Results: Across six institutions, a strong correlation between PM integration score and targeted therapy use was observed in metastatic non-small cell lung cancer (mNSCLC) (R^2^ = 0.81), an indication with a significant number of approved targeted agents. Conversely, a strong correlation between PM integration score and clinical trial enrollment was observed in metastatic triple-negative breast cancer (TNBC) (R^2^ = 0.63), an indication with fewer marketed targeted therapies but an active targeted therapy pipeline. Conclusion: The results in these cases suggest that PM integration is a strong indicator of high-quality care practices for both utilization of targeted therapy in more mature PM indications (e.g., mNSCLC) and clinical trial enrollment in more emerging PM indications (e.g., TNBC).

## 1. Introduction

Personalized medicine (PM) is an important and growing component of cancer care. Stakeholders increasingly recognize the value of predictive and prognostic biomarker testing to help inform prevention and treatment decisions that could lead to better patient outcomes and efficiencies in care delivery [1]. There are more than 100 US Food and Drug Administration (FDA)-approved targeted therapies, meaning those whose labels reference specific biological markers, available for use in eligible patients with cancer [2]. There are also more than 60,000 genetic testing products on the US market, and a significant proportion of those are oncology companion diagnostics [3]. Integrating personalized medicine into oncology practice, however, requires a shift from traditional “one-size-fits-all” policies and practices to an approach based on biomarker testing to better understand individual patient characteristics. A failure to make this shift is associated with clinical practice gaps that have disrupted the delivery of precision oncology. A recent study showed that only approximately 36% of patients with newly diagnosed metastatic non-small cell lung cancer (mNSCLC) are benefiting from precision oncology, indicating a significant clinical impact deficit [4]. Delivering precision oncology requires an investment in updated technologies, policies, practices, and associated workforce education. As oncology providers consider integrating PM strategies into their health care delivery systems, it is important that they have evidence showing that the approach improves clinical care.

To better understand how PM practices are being adopted in clinical settings, our group previously developed a novel, multifactorial framework to systematically evaluate the state of PM integration in a representative sample of health care delivery systems across the US [5]. This framework interrogated eight separate dimensions of PM integration to provide a holistic PM adoption fingerprint for each institution: (1) collection of genomic data, (2) collection of other omics data, (3) collection of non-laboratory data, (4) diagnostic testing guidance and accessibility, (5) data utilization, (6) data sharing, (7) internal funding, and (8) leadership support. The application of this framework revealed that PM integration levels fall across a spectrum and are widely distributed in terms of PM integration across clinical areas. While higher-ranked institutions are frequently thought of as outstanding health systems, there is a need for evidence linking personalized medicine integration to improved clinical care. A better understanding of the association between PM integration and improved health care delivery and clinical outcomes may help justify the adoption of the policy changes and investments necessary for broad-based PM implementation. Historically, it has been challenging to prove this association due to myriad factors, including the identification of appropriate metrics to assess care quality, the ability to accurately measure PM adoption, and access to data at the health system level to understand clinical performance. This study is unique in its ability to address these hurdles in order to gain insight into the impact of PM practices on quality of care.

In the context of PM, care quality is typically defined as high-value treatment that ensures the right patient is receiving the right treatment at the right time. Given the pace at which the oncology therapeutic landscape is evolving, high-value treatment could include either marketed targeted therapies or those being studied in clinical trials. Treatment options also vary by clinical area, and their appropriate delivery is impacted by the maturity of PM approaches. Therefore, evaluation of care quality in different indications may provide meaningful insights.

Here, we evaluate how care quality, specifically defined as the use of targeted therapies or enrollment in clinical trials, correlates with PM integration scores at six regional community health systems within three large health systems in the US. These health systems were selected in part because they met two criteria: (1) they have existing infrastructure to collect real-world data that provides insight into the care quality metrics of interest, and (2) they have the ability to provide the information required to evaluate the PM integration score.

This study focused on four clinical areas representing differing levels of maturity of the PM landscape: HER2+ metastatic breast cancer (mBC), HR+/HER2− mBC, HR−/HER2- mBC [also known as triple-negative breast cancer (TNBC)], and mNSCLC. In HER2+ mBC, testing awareness and practices are so widely entrenched that a PM approach is effectively the standard of care. Patients with HR+/HER2− mBC have fewer options for PM approaches, with only seven on-label therapies available for treatment [6]. Furthermore, less than 10% of clinical trials in mBC are focused within this segment of patients [7]. However, these patients also have the best prognosis of all mBC subtypes [6]. TNBC patients have even fewer opportunities to take advantage of on-label therapies; less than five approved treatments are available, and overall, only 35% of patients have actionable oncogenic mutations [8]. However, these patients have many more trial options, with almost 40% of mBC trials investigating treatments for TNBC [8]. In contrast, more than 50% of mNSCLC patients have actionable oncogenic driver mutations to guide targeted therapy use [9]. An extensive pool of more than 20 FDA-approved, biomarker test-driven-targeted therapeutic options is available for responder patients in mNSCLC [10].

## 2. Materials and Methods

To conduct this study, we obtained real-world data (RWD) on the utilization of targeted therapies and clinical trial enrollment from the Syapse^®^ Learning Health Network (LHN). The database integrates structured and manually curated patient data from electronic health records (EHRs), laboratory and radiology/imaging systems, computerized order entry systems, and facility cancer registries. To correlate PM integration scores with these data, we used the previously described framework to evaluate institutional PM scores at six selected organizations across three health systems that had a diverse representation of size, geographic location, and investment in PM initiatives (Table 1). The patient populations were relatively equivalent in terms of access to healthcare services, as indicated by the insurance status breakdown (Table 1). Because this study is only evaluating oncology-specific outcome measures, we used the institution’s oncology-specific PM integration score, as opposed to the institution’s overall score, to evaluate progress toward PM adoption.

The information needed to calculate the PM integration score for each institution was collected through an online survey. The survey respondents were key stakeholders in the Precision Medicine programs at their respective institutions and represented a variety of health system roles including: Cancer Service Line Director, Lab Director, Medical Director, Academic Officer and Precision Medicine Director. Each institution’s PM score was calculated using the same methodology as the previously described study [5]. In addition, we conducted follow-up interviews with each survey respondent to further clarify and adjust responses as needed to assure appropriate interpretation of survey questions within the context of oncology and PM.

Using the Syapse^®^ LHN, we analyzed the number of eligible patients with NSCLC or BC (HER2+, HR+/HER2−, and TNBC) diagnosed as metastatic from January 2017 through December 2022 (either de novo or at recurrence) at each of the six selected institutions. For each institution and population, we analyzed the proportion of patients who had received a targeted therapy or were enrolled in a clinical trial at any time following the diagnosis of metastatic disease, based on the RWD available as of August 2023. Targeted therapies included all FDA-approved therapies that were associated with a specific biomarker in the Syapse LHN drug dictionary, excluding immune checkpoint inhibitors (e.g., PD-L1) or hormonal therapy (e.g., selective estrogen receptor modulators, or aromatase inhibitors). Clinical trial drugs included all antineoplastic agents associated with a clinical trial flag in Syapse LHN datasets. An R-squared linear correlation analysis was then performed between the proportion of patients receiving targeted therapy or enrolled in a clinical trial and the institution-specific PM integration score. The level of correlation between these outcome measures and the PM integration score was determined.

## 3. Results

**Figure 1:**
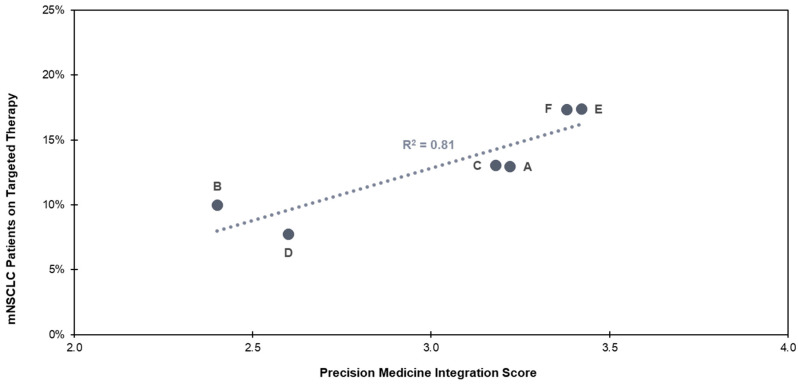
Targeted therapy use in mNSCLC for institutions A–F. Institution F has adopted the same protocol for all mNSCLC patients as institution E, and PM integration score has been adjusted to reflect this. For all other indications, the PM integration score for institution F is 2.5.

Within the six health care delivery institutions examined in this study, a higher PM integration score had a statistically significant and strong association (R^2^ = 0.81, *p* < 0.05) with a higher percentage of mNSCLC patients receiving targeted therapies (Figure 1). More than 15% of eligible mNSCLC patients receiving care at institutions E (integration score = 3.4) and F (integration score = 3.4) received appropriate targeted therapies, while less than 10% received appropriate targeted therapies at institutions D (integration score = 2.6) and B (integration score = 2.4).

**Figure 2:**
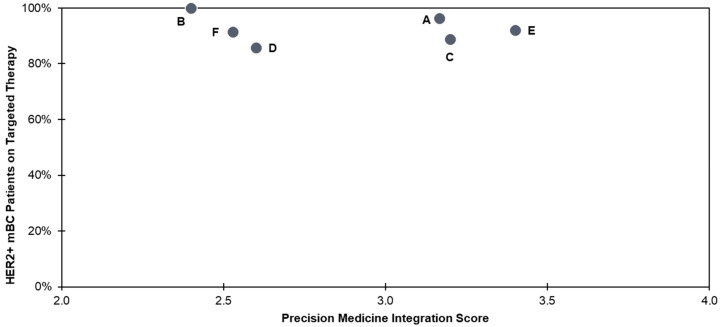
Targeted therapy use in HER2+ mBC for institutions A–F.

Notably, across all institutions, higher PM integration scores did not correlate with a higher percentage of HER2+ patients receiving the appropriate targeted therapies. Use of targeted therapies in HER2+ patients was high, ranging from 85–100% for all institutions (Figure 2). The consistent use of targeted therapies in HER2+ mBC suggests that the PM approach for this indication is so mature that it has become a mainstay of clinical practices across institutions.

The percentage of eligible HER2+ mBC patients who received targeted therapies (85–100%) was highest overall as compared to the maximum percentages of similarly treated patients with mNSCLC (17.4%), HR+/HER2− mBC (21.1%), and TNBC (36.0%) (Table 2). The strongest correlation of targeted therapy use and PM integration score was in mNSCLC (R2 = 0.81), compared to HR+/HER2− mBC and TNBC (R^2^ = 0.03 and R^2^ = 0.06, respectively). Notably, institution D (integration score = 2.6) was associated with a lower percentage of appropriate targeted therapy use in mNSCLC patients but relatively high levels of appropriate targeted therapy use in mBC patients, pointing to an inherent variability at select institutions of PM integration between cancer indications. This suggests that institution D may have a concerted focus on improving mBC care.

**Figure 3:**
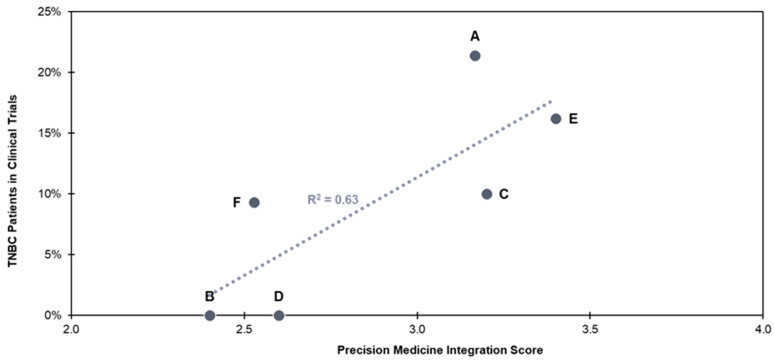
Clinical trial enrollment in TNBC for institutions A–F.

The percentage of TNBC patients who were enrolled in clinical trials after receiving biomarker testing correlated moderately with PM integration score but was not statistically significant (R^2^ = 0.63, *p* > 0.05) (Figure 3). Clinical trial enrollment may not be a strong indicator of PM integration for all indications, however. In contrast to TNBC, R^2^ values for trial enrollment of mNSCLC, HER2+ mBC, and HR+/HER2− mBC patients at these community hospital systems were very weak (R^2^ = 0.41, R^2^ = 0.22, and R^2^ = 0.003, respectively). In the case of mNSCLC and HER+ mBC, patients have positive biomarker test results that indicate the use of a well-established, high-value, approved targeted therapy and do not typically need to explore other treatment options. For HR+/HER2− mBC, PM activity is generally limited, possibly due to the overall better prognosis of these patients as compared to other mBC subtypes. Clinical trial enrollment may be associated more strongly with PM integration in cancer indications where PM approaches are less mature. In such instances, the status of various biomarkers can inform more potential targeted treatment options.

## 4. Discussion

In this study, we demonstrate that in a patient population with a broad and complex array of targeted therapy options (mNSCLC), increased PM integration scores correlated with higher utilization of targeted therapies. In contrast, in patient populations with either a single clear standard of care targeted therapy (HER2+ mBC) or fewer established targeted therapy options (mTNBC), there was no clear correlation between targeted therapy utilization and PM integration. We also saw significant correlation between targeted therapy use within clinical trials and PM integration in a patient population with few established targeted therapy options (mTNBC), but limited correlation in a patient population with an array of on-label therapy options.

Taken together, these two findings suggest that institutions with greater PM integration are succeeding in increasing access to targeted therapies via both approved and clinical trial therapies in a nuanced and clinically relevant manner. Rather than increasing utilization universally, we see the impact of PM integration in the populations and via the treatment modalities (approved therapy vs. clinical trial therapy) that are most relevant to the patient population. In the context of our definition of care quality (delivery of high-value treatment to individual patients based on their tumors’ specific biomarker profiles), our results suggest that increased PM integration correlates with improved clinical care. Delivering targeted treatments should lead to individual patient benefits involving improved outcomes, reduced side effects, and greater patient satisfaction [11]. Health system benefits should include improved efficiency and, potentially, reduced costs associated with reduced progression of disease [11].

While increased delivery of high-value targeted treatments was shown to be associated with institutions that score highly on the PM integration scale, it was also impacted by the availability of a diverse set of treatment options within an indication and the maturity of a PM approach. With mNSCLC, the institutions with higher integration scores may be better equipped to deliver appropriate targeted therapies for this indication, as it correlates with how well an organization is embracing complex and rapidly evolving PM paradigms. However, with mBC, whether HER2+ mBC, HR+/HER2− mBC, or TNBC, fewer available targeted therapy options may mitigate the impact of PM approaches in driving differential targeted therapy adoption. Furthermore, with HER2+ mBC, the precision oncology approach has matured to a point where biomarker testing to drive treatment with HER2 targeted therapies may be standard practice regardless of health care institution. In this context, HER2+ mBC should serve as an example indication for which efforts to deliver precision oncology broadly should strive. By investing in the personalized medicine approach across cancer types and increasing PM integration scores, health systems can more rapidly implement biomarker testing-driven, high-value personalized care in routine practice, as has been attained in HER2+ mBC.

There is a moderate correlation between integration score and clinical trial enrollment for TNBC. While the correlation coefficient was not strong, the results point to trends that may indicate improved care delivery for advanced cancer patients for which approved high-value treatment options may not be available but for which additional potential treatment options are being developed. We note that programs to enroll patients in clinical trials are sometimes complex and can vary between institutions [12], thereby leading to challenges in measuring clinical trial participation endpoints. However, the trend observed for TNBC, in which multiple biomarkers can inform treatment, is still notable. By contrast, the correlation was not strong in mNSCLC, HER2+ mBC, or HR+/HER2− mBC. For these biomarker-specific indications, the use of approved high-value targeted therapies in responder populations is more firmly entrenched. It is therefore likely that fewer patients explore clinical trial opportunities.

Our analysis is subject to various limitations. We draw our conclusions based on only a small number of institutions (six). While this provides real-world evidence of trends at these six institutions, it does not necessarily reflect the trends of different cohorts of institutions or across individual health systems. While the six institutions that were included in this study showed variability in PM integration, it should be noted that in order to be able to participate in the study, they had to have invested in building a real-world data capability with data sharing across institutions, as this is how the data was sourced. This aspect of study design was necessary to enable the study to be conducted using the Syapse LHN dataset but limited the range of systems that could be assessed. A larger cohort of institutions that represent different types of health care delivery institutions with differing levels of investment in data to support PM in oncology should be studied to make more definitive conclusions relevant to health systems overall. PM integration scores were determined based on an institutional survey. Responses are subject to interpretation of survey questions, and, therefore, integration scores may vary depending on the survey respondent. The six institutions involved in this study were community health systems; therefore, the academic health center perspective is not represented. This can impact integration scoring and endpoint measurement, especially related to clinical trial availability and enrollment. Measuring relevant clinical trial enrollment can be challenging due to variable and sometimes unclear clinical trial practices at an institution, but measurement is likely further mitigated by a lack of academic health centers within the cohort that are more active in clinical trial research.

The results suggest that greater PM integration leads to increased relevant targeted therapy use and clinical trial enrollment. These factors are considered to be indicators of greater care quality within precision oncology practice. Additional endpoints that can help demonstrate improved care include long-term outcomes and patient-reported satisfaction. Continuing this analysis to include a larger set of institutions, as well as exploring additional quality metrics and outcomes, will be important to further justify institutional investments in PM delivery infrastructure.

## 5. Conclusions

This analysis provides a view into the impact of adopting PM strategies on clinical care in oncology in a select set of institutions. By integrating PM strategies into their oncology care workstreams, health care delivery institutions are able to deliver more effective treatments and provide more treatment options to their cancer patients. While this is shown in mNSCLC and TNBC, it is likely reflective of other cancer types for which the status of multiple biomarkers can help inform care. The results of this study may help justify the adoption of policy changes and systemic investments associated with the implementation of PM strategies for health care institutions that are considering how to improve their oncology care. This, in turn, can help to reduce key barriers to PM adoption and broaden access to higher-quality care for patients.

## Figures and Tables

**Table 1:** Respondent demographics.

	Regional Health System 1 Institutions A, B, C	Regional Health System 2 Institution D	Regional Health System 3 Institutions E, F
Description	One of the top five nonprofit health systems in the US	Large regional health care system	One of the top five nonprofit health systems in the US
Regions covered	Midwest and Northeast	Mid-Atlantic	Midwest
Annual cancer patient volume	~43,000	~1000	~19,000
Breast and lung cancer patient insurance status	Private: 35%	Private: 36%	Private: 35%
	Medicare: 51%	Medicare: 52%	Medicare: 52%
	Medicaid: 6%	Medicaid: 4%	Medicaid: 7%
	Other: 6%	Other: 6%	Other: 5%
	Unknown: 2%	Unknown: 2%	Unknown: 1%
Cancer program details	Commission on Cancer accreditation Established a PM taskforce	Commission on Cancer accreditation Affiliate of a large academic hospital Established oncology precision medicine program in 2020	Commission on Cancer accreditation Established oncology precision medicine program in 2016

**Table 2:** Targeted therapy prescribing rates in mNSCLC, HR+/HER2− mBC, and TNBC for institutions A–F.

Institution PM Score	Share of Eligible Patients Prescribed Targeted Therapies			
	mNSCLC	HR+/HER2− mBC	TNBC	HER2+ mBC
A (3.2)	13%	9%	29%	96.3%
B (2.4)	10%	9%	0%	100.0%
C (3.2)	13%	8%	10%	88.9%
D (2.6)	8%	19%	36%	85.7%
E (3.4)	17%	16%	27%	92.0%
F (3.4)	17%	16%	25%	91.5%

## Data Availability

The original contributions presented in the study are included in the article, further inquiries can be directed to the corresponding author.
